# Aging Effect on Neurotrophic Activity of Human Mesenchymal Stem Cells

**DOI:** 10.1371/journal.pone.0045052

**Published:** 2012-09-17

**Authors:** Maria Brohlin, Paul J. Kingham, Liudmila N. Novikova, Lev N. Novikov, Mikael Wiberg

**Affiliations:** 1 Department of Integrative Medical Biology, Section of Anatomy, Umeå University, Umeå, Sweden; 2 Department of Surgical and Perioperative Sciences, Section of Hand and Plastic Surgery, Umeå University, Umeå, Sweden; University of Medicine and Dentistry of New Jersey, United States of America

## Abstract

Clinical efficacy of stem cells for nerve repair is likely to be influenced by issues including donor age and *in vitro* expansion time. We isolated human mesenchymal stem cells (MSC) from bone marrow of young (16–18 years) and old (67–75 years) donors and analyzed their capacity to differentiate and promote neurite outgrowth from dorsal root ganglia (DRG) neurons. Treatment of MSC with growth factors (forskolin, basic fibroblast growth factor, platelet derived growth factor-AA and glial growth factor-2) induced protein expression of the glial cell marker S100 in cultures from young but not old donors. MSC expressed various neurotrophic factor mRNA transcripts. Growth factor treatment enhanced the levels of BDNF and VEGF transcripts with corresponding increases in protein release in both donor cell groups. MSC in co-culture with DRG neurons significantly enhanced total neurite length which, in the case of young but not old donors, was further potentiated by treatment of the MSC with the growth factors. Stem cells from young donors maintained their proliferation rate over a time course of 9 weeks whereas those from the old donors showed increased population doubling times. MSC from young donors, differentiated with growth factors after long-term culture, maintained their ability to enhance neurite outgrowth of DRG. Therefore, MSC isolated from young donors are likely to be a favourable cell source for nerve repair.

## Introduction

Peripheral nerve injuries often result in lifelong reduced motor functions and minimal sensory recovery. Typically a gap nerve injury is treated with an autologous nerve graft but these are far from ideal since they involve sacrifice of another nerve with subsequent loss of sensation at the donor site [1]. Peripheral nerve grafts have also been to used to bridge experimental central nervous system defects allowing the in-growth of a limited number of axon pathways [2]. Currently an active area of research is the search for alternatives to the nerve graft such as artificial conduits constructed from natural or biosynthetic materials loaded with various types of regenerative cells [3], [4].

Schwann cells are the key cells in the peripheral nervous system providing the nervous tissue with cell adhesion molecules and various growth factors following injury [5]. For this reason a number of studies have investigated nerve regeneration following transplantation of Schwann cells [6]–[9]. However, Schwann cells are likely to have limited clinical application due to inadequate availability of nerve donor tissue and their slow growth rates *in vitro* [10], [11] so recently it has been suggested that stem cell transplants could be an alternative. Mesenchymal stem cells (MSC) can be accessed from various tissues such as bone marrow and adipose tissue and they have high plasticity and are capable to differentiate into many functional cell types [12]–[14]. Furthermore MSC express a wide variety of growth factors [15], [16] which could promote nerve regeneration. Recently we and others showed that glial-like differentiated mesenchymal stem cells could be a good substitute for autologous Schwann cells since they show functional and phenotypical identity with them [17]–[19].

From a clinical point of view it is of interest to identify the best source of cells in order to effectively provide enough cells to treat nerve injuries. Patient donor age influences the ability to obtain sufficient quantities of cells [20], [21] and aging in culture, with prolonged passaging, can result in senescence and influence proliferation and differentiation rates [22]. In this study we have investigated the expression of neurotrophic factors in MSC isolated from young and old donors (at early and late passage) and determined whether differentiation affects their ability to enhance neurite outgrowth *in vitro*.

## Materials and Methods

### Culture of Bone Marrow Mesenchymal Stem Cells (MSC)

Samples of human bone marrow were obtained from the iliac crests of six healthy donors during reconstructive surgery. The purpose, nature, and potential risks of the study were explained to the patients and informed written consent was obtained from the adult patients or from next of kin in the case of the minors. The donors were designated as follows: young donors (n = 3, 16–18 year, mean 17.3 years) and old donors (n = 3, 67–75 year, mean 73.7 years). Procedures were approved by the Local Ethical Committee for Clinical Research in Umeå University (no 03–425). Modification of a previously described protocol [10] was used to isolate and prepare primary cultures of MSC. Briefly, bone marrow samples were rinsed thoroughly with alpha Modified Eagle’s Medium (αMEM) containing 10% (v/v) foetal bovine serum (FBS) and 1% (v/v) penicillin-streptomycin (all from Invitrogen Life Technologies, Sweden). The cell suspension was centrifuged at 400 g for 5 min and the cell pellet was filtered through a 70 µm nylon mesh (BD Falcon, Becton Dickinson and Company, UK) and plated in 75 cm^2^ tissue culture flasks (Nunc, USA) and incubated at 37°C, 5% (v/v) CO_2_. After 12–24 h in culture, the supernatant containing non-adherent cells was removed and discarded and fresh medium added. The cells attached to the culture flask were cultured (37°C, 5% CO_2_) for 2–3 weeks with medium changes every 48 h. When the cultures had reached 80% confluence, the cells were enzymatically detached from the flask using 0.25% trypsin/EDTA solution (Invitrogen Life Technologies) and re-seeded in new culture flasks at a density of 5×10^3^ cells per cm^2^.

At passage 2 the undifferentiated cells were seeded at a density of 5×10^3^ cells per cm^2^ and cultured for one week (passage 3). The cells were counted and reseeded at the same density and cultured for another week. This was repeated until passage 11. From the cell counts, population doubling (PD) times were calculated.

**Figure 1:**
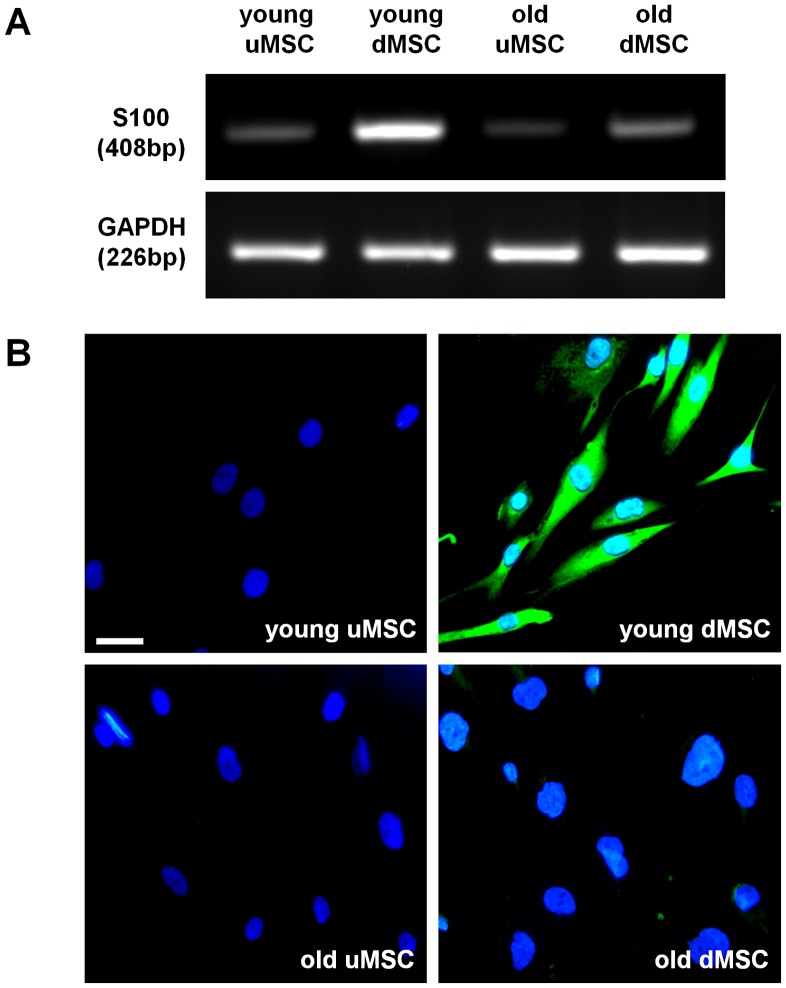
S100 expression in differentiated mesenchymal stem cells. (**A**) RT-PCR analysis of S100 from undifferentiated MSC (uMSC) and differentiated MSC (dMSC) harvested from young and old donors at early passage. The amplicons (size shown in base pairs; bp) were resolved on a 2% agarose gel and stained with Gel Red dye. (**B**) Cell cultures were stained for S100 protein (green) and the nuclei counter-stained with DAPI (blue).

### Differentiation

The differentiation process was initiated in sub-confluent MSC at the second and eleventh passage by treatment with medium supplemented with 1 mM β-mercaptoethanol (Sigma-Aldrich, Sweden) for 24 h. Cells were then incubated for 72 h with growth medium containing 35 ng**/**ml all-*trans*-retinoic acid (Sigma-Aldrich) followed by growth medium supplemented with 5.7 µg/ml forskolin (MP Biomedicals, Sweden), 10 ng/ml basic fibroblast growth factor (bFGF; Invitrogen), 5 ng/ml platelet derived growth factor–AA (PDGF-AA; Millipore, UK) and 126 ng/ml glial growth factor-2 (GGF-2; a gift from Acorda Therapeutics Inc, USA). The cells were maintained in this supplemented medium for two weeks with medium changes every 72 h to establish differentiated cultures [17], [18]. Cells were then stained with S100 antibody (polyclonal, 1∶100, Dako Sweden) as previously described [17]. Undifferentiated cells were cultured in parallel with no passage in order to maintain the same culture conditions. Cells were then trypsinised and analysed at passage 3 and 12.

### Dorsal Root Ganglia Co-culture

Dorsal root ganglia (DRG) were harvested from the spinal cords of adult female Sprague-Dawley rats using a previously described protocol [17], [18], [23]. All experiments involving the use of animals were approved by the Northern Swedish Committee for Ethics in Animal Experiment and in compliance with the European Communities Council Directives (86/609/EEC). Dissociated neurons were resuspended in Neurobasal medium with B12 supplement (Invitrogen). The DRG neurons were seeded onto laminin-coated glass cover slips (Sigma-Aldrich) inserted into 6 or 24-well plates and incubated for 24 h (37°C, 5% CO_2_). In another series of experiments, the SH-SY5Y human neuronal cell line (ECACC/Health Protection Agency Culture Collections, UK) was seeded in α-MEM medium at a density of 5000 cells onto coverslips and allowed to settle for 24 h. Cultures of undifferentiated (uMSC) and differentiated (dMSC) stem cells from the three donors in each group were seeded onto 1.0 µm pore size polyethylene terephthalate membrane cell culture inserts (BD Falcon) at a density of 75,000 cells/ml and incubated for 24 h (37°C, 5% CO_2_). The inserts were then checked for cell adherence, then placed into wells containing DRG neurons or SH-SY5Y cells to establish the co-culture; these were allowed to incubate together for additional 24 h (37°C, 5% CO_2_). Additional controls with the cell-free inserts containing stem cell medium only were also analyzed. Other inserts contained either 10 µg/ml anti-BDNF antibody (Millipore) or 1 µg/ml anti-VEGF antibody (R&D Systems, UK). In another series of experiments the conditioned medium was removed from inserts containing the stem cells (plated as above) and transferred to the DRG neuron cultures. After 24 h in co-culture with stem cells in inserts or in the presence of conditioned medium, the neurons on the cover slips were fixed in 4% (w/v) paraformaldehyde (20 min, 4°C) and immunostained for anti-βIII tubulin (monoclonal, 1∶500, Sigma-Aldrich) with secondary antibody Alexa Fluor 488 goat anti-mouse IgG (1∶1000, Invitrogen). The cover slips were mounted with Prolong™ for Fluorescence (Invitrogen) and examined using a fluorescence microscope. The images were captured for quantification at ×10 magnification using an Evolution QEi monochrome digital camera; image analysis was performed using Neurolucida (MBF Bioscience, USA). All neurites were traced to generate total neurite outgrowth data (in µ m). Three independent co-culture experiments were carried out for neurite outgrowth assessment.

**Figure 2:**
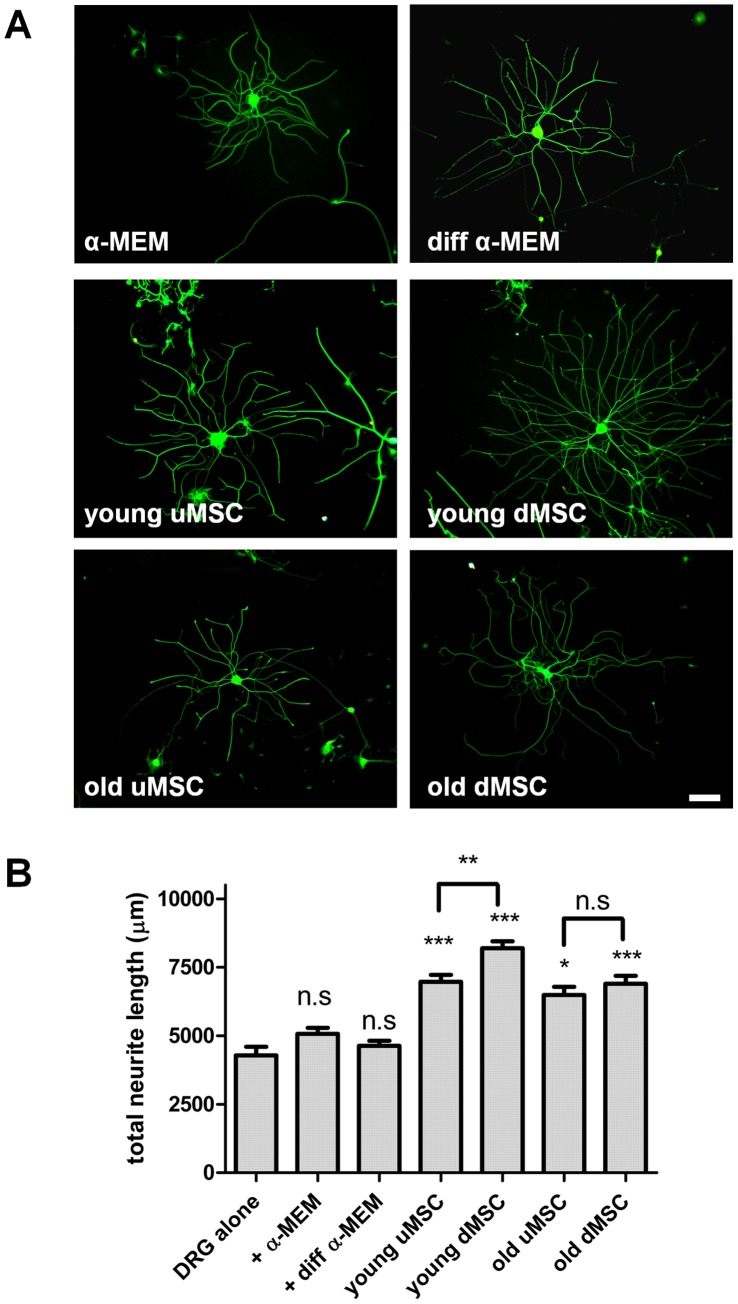
Mesenchymal stem cells enhance neurite outgrowth of co-cultured DRG neurons. (**A**) Immunocytochemical staining for βIII tubulin (Alexa Fluor 488) visualized neurite outgrowth from DRG following 24 h co-culture with medium only controls (α-MEM and diff α-MEM) and undifferentiated MSC (uMSC) and differentiated MSC (dMSC) from young and old donors (T3) seeded in tissue culture inserts above the neurons. (**B**) Quantitative analysis of total neurite outgrowth in the co-cultures. ***P<0.001 significantly different to respective media only controls. n.s not significantly different (α-MEM and diff α-MEM versus DRG alone and dMSC versus uMSC old donors).

### RT-PCR for Neurotrophic Factors

The RNeasy™ mini kit (Qiagen Nordic, Sweden) was used according to the manufacturer’s protocol for the isolation of total RNA from the cell pellets of the undifferentiated MSC (uMSC) and differentiated MSC (dMSC) (cultured under identical conditions to those used for neurite analysis) and then 1 ng RNA was incorporated into the One-Step RT-PCR kit (Qiagen) per reaction mix. Primers were manufactured by Sigma, UK (Table S1). A thermocycler (Biometra, Germany) was used with the following parameters: a reverse transcription step (50°C, 30 min), a nucleic acid denaturation/reverse transcriptase inactivation step (95°C, 15 min) followed by cycles of denaturation (95°C, 30sec) and annealing (30sec, optimised per primer set as described in Table S1) and primer extension (72°C, 1 min) followed by final extension incubation (72°C, 5 min). PCR amplicons were electrophoresed (50 V, 90 min) through a 1.5% (w/v) agarose gel and the size of the PCR products estimated using Hyperladder IV (Bioline, UK). Samples were visualised under UV illumination following GelRed™ nucleic acid stain (BioNuclear, Sweden) incorporation into the agarose.

### Enzyme-linked Immunosorbant Assay (ELISA)

uMSC and dMSC were seeded at an identical density to those used for neurite analysis; 15000 cells per 200 µl media and maintained for 48 h. The supernatant from the cells was then collected and analysed by ELISA using the ChemiKine™ Brain Derived Neurotrophic Factor (BDNF) sandwich ELISA kits (Chemicon, UK) or Vascular Endothelial Growth Factor (VEGF) sandwich ELISA kit (RayBio, GA, USA) according to the manufacturer’s protocol. All samples were analysed in triplicate and the absorbance was measured at 450 nm (Spectra Max 190 microplate reader (Molecular Device, USA).

### Statistical Analysis

Statistical analysis was conducted using GraphPad Prism software (GraphPad Software Inc., USA). The data for neurite outgrowth quantification are expressed as mean ± SEM and analysed by t-test or one-way ANOVA with Newman Kreuls post hoc test where appropriate. Statistical significance was determined as *P<0.05, **P<0.01, ***P<0.001.

## Results

### MSC Differentiation

Stem cells from young or old donors were treated with a mixture of glia growth factors in order to differentiate them towards a glial cell phenotype as previously described by us [17]. We found that S100 gene expression was up-regulated in the young donor cells but was markedly lower in the cells taken from old donors (Fig. 1A). Consistent with this we were able to detect S100 protein in the differentiated young donor cells but not in old donor cultures (Fig. 1B).

**Figure 3:**
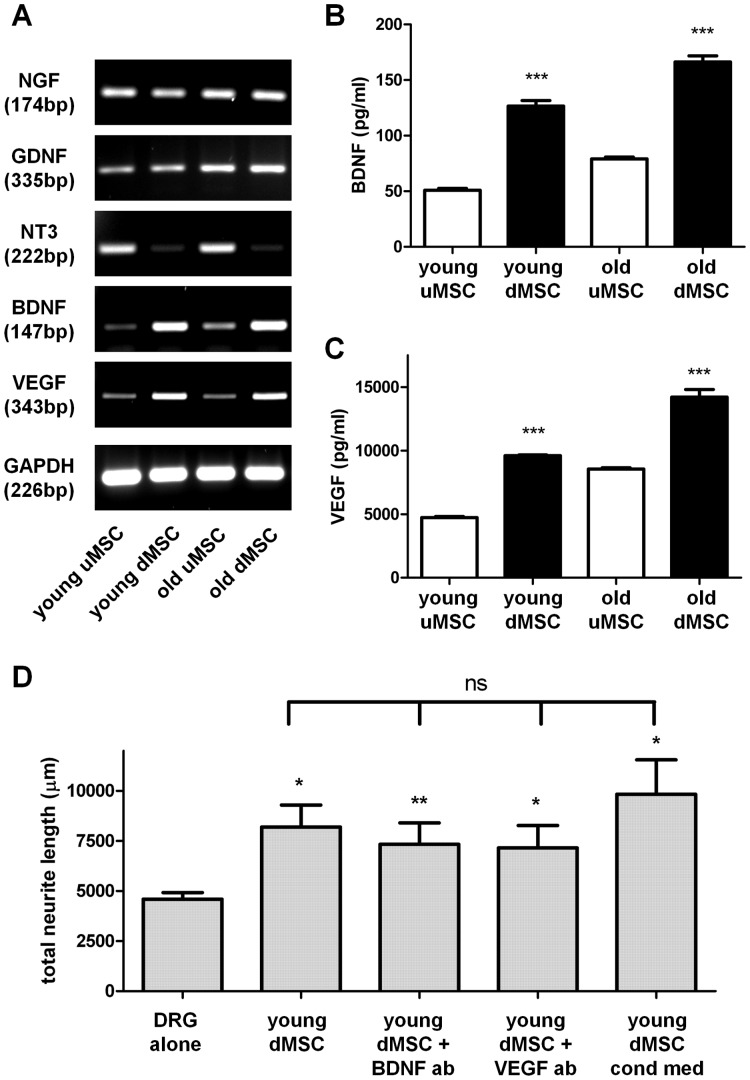
Neurotrophic factor expression in mesenchymal stem cells. (**A**) RT-PCR analysis of neurotrophic factor transcripts NGF, GDNF, NT-3, BDNF, VEGF from undifferentiated MSC (uMSC) and differentiated MSC (dMSC) harvested from young and old donors. Amplicon size is shown in base pairs (bp). (**B** and **C**) ELISA was used to determine BDNF and VEGF protein levels in cell culture supernatants. ***P<0.001 differentiated MSC significantly higher compared with undifferentiated MSC. (**D**) Neurite outgrowth of DRG neurons after 24 h in the absence (DRG alone) or presence of young donor dMSC alone or with BDNF and VEGF blocking antibodies (ab) or when treated with conditioned medium (cond med) prepared from dMSC. *P<0.05; **P<0.01 significantly different from DRG alone; ns, no significant difference between groups.

### Neurite Outgrowth – Young Versus Old Donors

Next we established co-cultures of early passage human MSC, either undifferentiated (uMSC) or differentiated (dMSC), with rat dorsal rat ganglia (DRG) neurons. The neurite outgrowth evoked by the stem cells was detected by immunocytochemical staining for βIII tubulin (Fig. 2A). Compared with their respective media only (negative controls) both uMSC and dMSC enhanced neurite outgrowth of the DRG neurons (Fig. 2A). It also appeared that differentiated cells taken from young donors generated the most neurite outgrowth (Fig. 2A). These observations were confirmed by using quantitative computerized image analysis of total neurite length (Fig. 2B). Neither growth medium nor differentiation medium alone significantly affected total neurite length compared with DRG neurons grown alone without tissue culture inserts. Both young and old donor uMSC significantly (P<0.001 and P<0.05 respectively) enhanced total neurite length. Differentiation of young donor MSC produced the greatest total neurite length (8195±254.3 µm) and significantly enhanced the levels above the undifferentiated cells (uMSC  = 6967±252.2 µm, P<0.01). In contrast differentiated old donor MSC showed no significant difference from undifferentiated old donor MSC (6492±295.7 µm and 6898±292.3 µm respectively).

### Trophic Factor Expression Levels

To investigate the possible growth factors responsible for the enhanced neurite outgrowth of DRG neurons we measured the trophic factor gene transcripts in MSC using semi-qualitative RT-PCR with GAPDH as a house-keeping control gene (Fig. 3A). Undifferentiated MSC (passage 3) from both young and old donors expressed transcripts for nerve growth factor (NGF), glial derived neurotrophic factor (GDNF), neurotrophin-3 (NT-3), brain derived neurotrophic factor (BDNF) and vascular endothelial growth factor (VEGF), all molecules which have been reported to promote neurite outgrowth from DRG neurons. Differentiation of MSC had no effect on the levels of NGF or GDNF but resulted in the down-regulation of NT-3 transcript. In contrast, both BDNF and VEGF transcript levels were elevated by the differentiation process (Fig. 3A). To confirm the gene expression changes led to enhanced translation and production of protein we performed ELISA on the cell supernatants. Both BDNF and VEGF were expressed to a higher extent in the dMSC compared with uMSC (p<0.001) in young and old donors (Figs 3B and C). To determine any role for BDNF and VEGF in the MSC mediated neurite outgrowth we performed DRG neurite outgrowth experiments in the presence of anti-BDNF and anti-VEGF blocking/neutralizing antibodies (Fig. 3D). Differentiated MSC from young donors significantly enhanced DRG total neurite length (*P<0.05) but this was not significantly altered in the presence of either antibody (Fig. 3D). However, conditioned medium from differentiated MSC was as effective for inducing neurite outgrowth as co-culture with live differentiated MSC (Fig. 3D) suggesting that other soluble, released growth factors were responsible for the effects observed.

**Figure 4:**
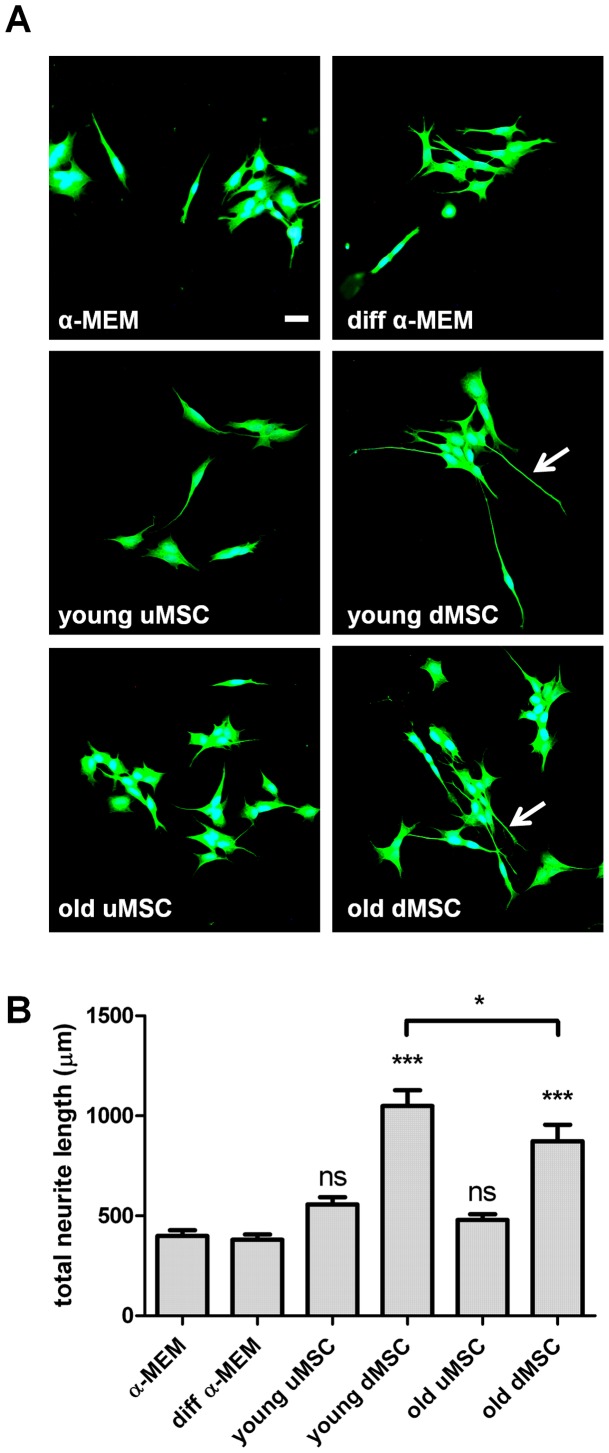
Mesenchymal stem cells enhance neurite outgrowth from the human SH-SY5Y neuronal cell line. (**A**) Immunocytochemical staining for βIII tubulin (Alexa Fluor 488) visualized neurite outgrowth (highlighted with arrows) from SH-SY5Y cells following 24 h co-culture with medium only controls (α-MEM and diff α-MEM) and undifferentiated MSC (uMSC) and differentiated MSC (dMSC) from young and old donors seeded in tissue culture inserts above the neurons. (**B**) Quantitative analysis of total neurite outgrowth in the co-cultures. ***P<0.001 significantly different to respective media only controls; *P<0.05 significantly different young dMSC versus old dMSC; ns not significantly different to respective media only controls.

Since MSC have been shown to exert immune enhancing functions and the DRG neuron co-culture model was xenogeic, we repeated the studies with a human neuronal cell line to ensure our results were not due to a xenogenic effect. Co-culture of differentiated MSC with SH-SY5Y neurons resulted in enhanced neurite outgrowth from the human cells (Fig. 4), replicating the results from the rat DRG neuron studies. Furthermore, the neurite outgrowth evoked from old donor dMSC was significantly less than that mediated by young donor dMSC (Fig. 4B).

### Cell Morphology and Proliferation – Effect of Passage

**Figure 5:**
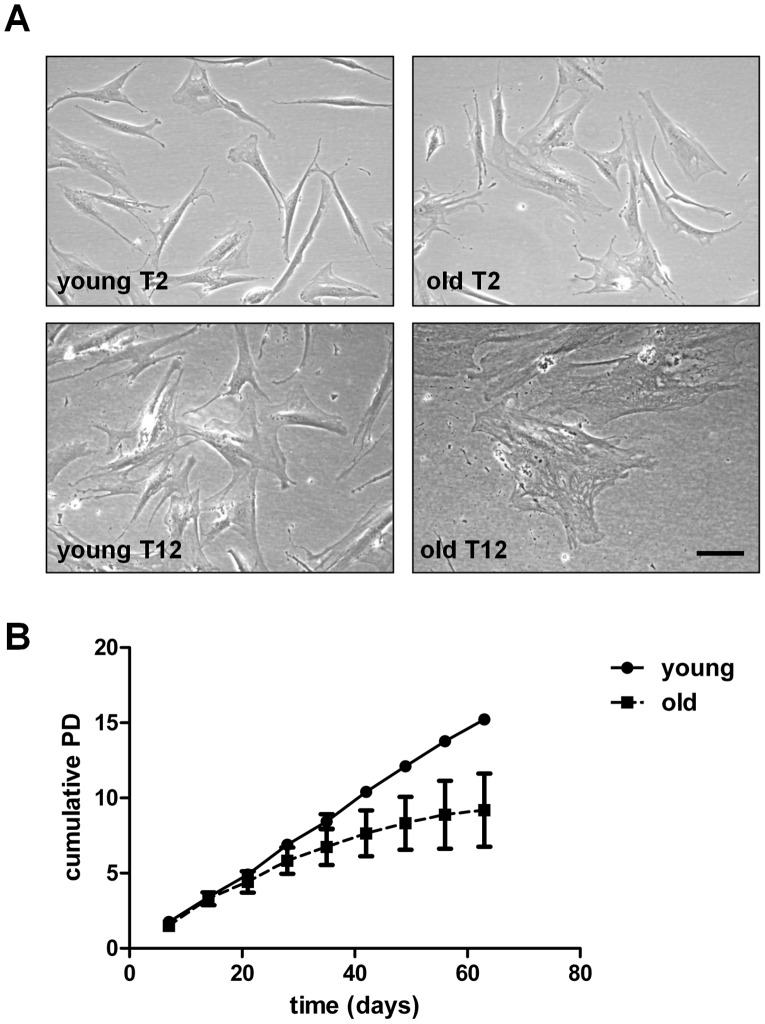
Change in undifferentiated stem cell morphology and proliferation after time in culture. (**A**) Undifferentiated MSC showed spindle shaped morphology at T2 but cells from old donors show a bigger more flattered shape at T12. **(B**) uMSC were cultured for nine weeks and passaged and counted on a weekly basis. Cumulative population doublings were calculated and plotted against time.

Our undifferentiated cell cultures showed a classical fibroblast-like morphology at early passage (T2) from both young and old donors (Fig. 5A). After time in culture the old donor MSC adopted a noticeably bigger flattered shape compared with the young donor MSC. Culturing the undifferentiated cells over a time period of ten weeks (T2–T11) showed a progressively decreased proliferation rate for the old donors compared with the young donors. Cumulative population doublings (PD) showed a continual linear trend for young donor MSC whereas the old donor cells began to plateau after 5 passages (Fig. 5B).

### Neurite Outgrowth – Effect of Passage

**Figure 6:**
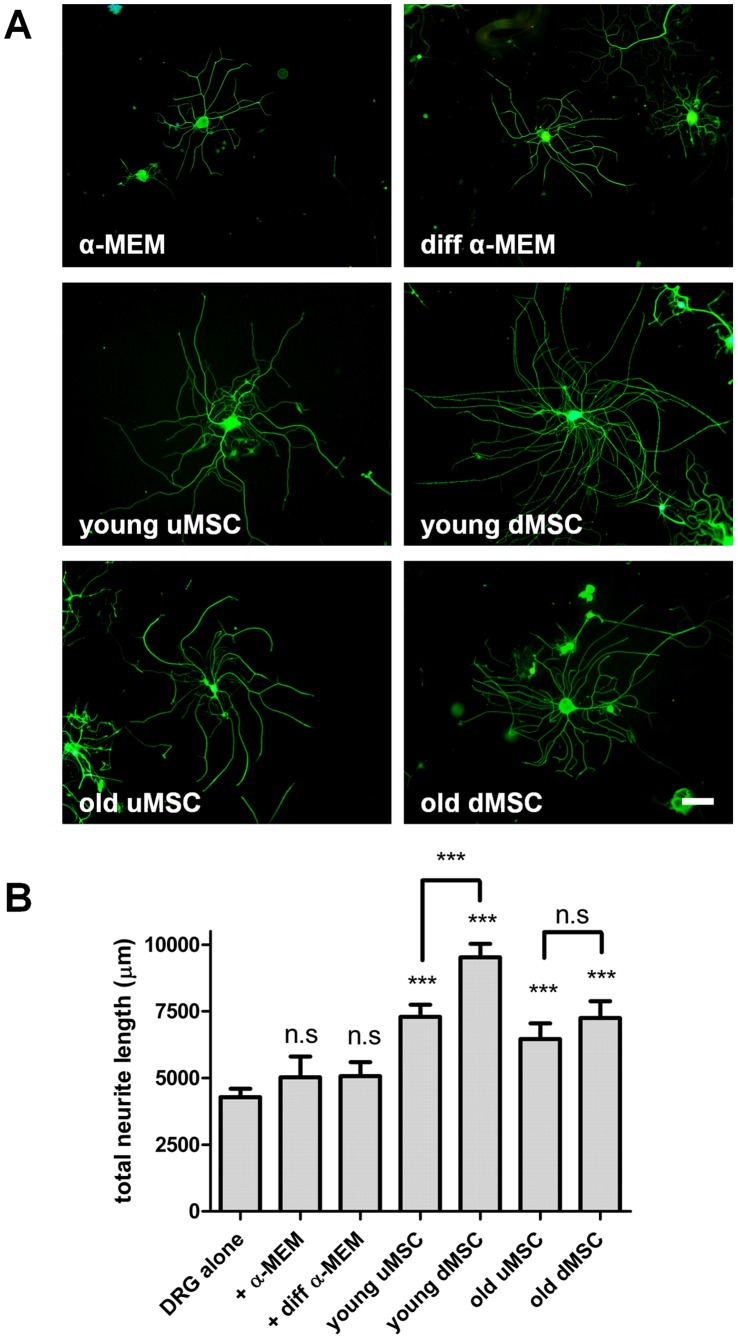
Late passage mesenchymal stem cells enhance neurite outgrowth of co-cultured DRG neurons. (**A**) Immunocytochemical staining for βIII tubulin (Alexa Fluor 488) visualized neurite outgrowth from DRG following 24 h co-culture with medium only controls (α-MEM and diff α-MEM) and undifferentiated MSC (uMSC) and differentiated MSC (dMSC) from young and old donors (T12) seeded in tissue culture inserts above the neurons. (**B**) Quantitative analysis of total neurite outgrowth in the co-cultures. ***P<0.001 significantly different to respective media only controls. n.s not significantly different (α-MEM and diff α-MEM versus DRG alone and dMSC versus uMSC old donors).

Co-cultures between late passage MSC and DRG neurons were also established (Fig. 6). The trend for neurite outgrowth mirrored that observed with the cells used at passage 3 (Fig. 2). All cell types studied evoked superior neurite outgrowth compared with their respective media only negative controls (P<0.001). Furthermore, the enhanced effect of differentiated cells from the young donors was maintained at these late passages (uMSC  = 7295±456.1 µm, dMSC  = 9523±505 µm total neurite length; P<0.001).

## Discussion

We have previously shown that human MSC can be differentiated to express a number of glial cell markers such as S100 and GFAP and that these cells promote neurite outgrowth [17]. In this study we have investigated the effect of patient donor age on these properties. Weak S100 mRNA expression levels were observed in undifferentiated MSC from young and old donors but the S100 protein was not detectable by immunocytochemistry. It is possible that the mRNA is subject to post transcriptional regulation preventing translation to protein or alternatively the protein expression levels were below our limit of detection. However, importantly we found that both S100 mRNA and protein levels were increased upon differentiation of young donor MSC but this was not the case for old donor cells. A recent study similar to ours also showed that neuroectodermal differentiation of human MSC is limited in old donors (>45 years) and that the levels of neural stem cell markers, nestin and neuroD, were lower in old donors [24]. In contrast, chondrocyte differentiation of the MSCs was unaffected by age. Another study by the same group showed that a panel of neurotrophic factors was up-regulated by the neuroectodermal differentiation protocol [25] but they did not study the effect of age on this process. We also assessed the neurotrophic factor profile of MSC prepared from young and old donors; before and after treatment with the glial growth factors, bFGF, PDGF-AA, GGF-2 and forskolin. We further investigated their functional effect in a bioassay with neurons.

MSC from young and old donors at early passage (T3) significantly enhanced neurite outgrowth of DRG neurons, most likely as a result of secretion of soluble neurotrophic factors. We found mRNA transcripts for NGF, GDNF, NT3, BDNF and VEGF expressed in undifferentiated MSC. In a previous study, a screen of a human MSC cDNA library also revealed transcripts for BDNF and NGF but not NT-3 [26]. Interestingly, expression of BDNF and NGF proteins were restricted to sub-populations of MSC indicating heterogeneity of the cultures which could possibly account for our ability to detect NT-3 transcripts in our cultures. When we treated the human MSC with the glial cell growth factors we found BDNF transcript expression was enhanced and a similar effect on BDNF protein levels was confirmed by ELISA. This result is consistent with previous studies in rat MSC [27]. BDNF levels correlate with enhancement of SH-SY5Y [26] and DRG [23] neurite outgrowth in response to MSC. However, our study showed that BDNF levels were increased in both young and old donor MSCs treated with glial growth factors, whereas neurite outgrowth was only further enhanced by young donor MSC treated in this manner. Furthermore, addition of an anti-BDNF blocking antibody to the MSC did not significantly reduce the neurite outgrowth. This likely suggests that some other growth factors have an overriding effect on differentiated MSC evoked neurite outgrowth. Expression of VEGF in MSC has been extensively documented from the viewpoint of the angiogenic capacity of the cells. However, more recently it was shown that MSC treated with a similar mix of glial growth factors as used in our study, could increase levels of VEGF, which was directly correlated with neurite outgrowth [28]. VEGF blocking antibodies also reduced the positive effect of MSC on axonal outgrowth of spinal nerves in a demyelinating organotypic spinal cord slice culture [28]. VEGF secreted by adipose derived stem cells can also protect cultured neurons against glutamate evoked excitoxicity [29]. We found that VEGF was up-regulated by the differentiation process, but as with BDNF, this response was also observed in old donor MSCs and furthermore anti-VEGF antibody did not reduce neurite outgrowth.

The MSC effects were not specific to rat DRG neurons because when co-cultured with human SH-SY5Y neurons, old donor differentiated MSC evoked significantly less neurite outgrowth than from young donor differentiated MSC. We conclude that MSC from both young and old donors express significant levels of neurotrophic factors but that some yet to be identified molecules are not produced (or at significantly lower levels) during the differentiation process in old donor MSC. Alternatively some growth inhibitory molecules produced by old donor cells block the effects of the neurotrophic factors. One way to further determine the differences between young and old donors could be to perform a mixed competitive assay, using different ratios of young:old donor cells (e.g. 1∶1, 1∶2, 1∶4, 1∶8, 1∶16) in co-culture with the neurons. In this way, the relative ability of each donor cell population to enhance neurite outgrowth would be determined, enabling a better understanding of the individual neurotrophic phenotypes of the cells. With recent advances in proteomic analysis, in our future studies it might also be possible to characterize new human proteins which modulate neurite outgrowth. This would enable greater understanding of the molecular pathways mediating interactions with neurons and identify new therapeutic targets for treatment of neurological diseases. Recent studies have characterized the cell surface proteome of human MSC which will allow better characterisation of sub-populations of stem cells derived from different tissues [30]. Another study showed that human bone marrow MSC derived from different donors had very similar proteomic expression profiles [31]. Proteomic analysis has also been used to study changes in protein expression occurring as a result of differentiation of human MSC towards the osteogenic lineage [32]. Perhaps of more specific relevance to our work is secretome proteomics, which aims to characterize the complex mix of molecules secreted from living cells. Recent work using these techniques has also identified the molecules secreted during osteogenic differentiation [33]. To our knowledge there are no published in-depth analyses of the human MSC secretome during neuro/glial cell differentiation.

MSC from old donors show reduced levels of the proliferation marker ki67 suggestive of a reduced growth rate [24]. In our study we calculated the population doubling times by passaging, counting and re-plating a defined number of cells every week up to 9 weeks. We found that cells from young donors maintained their proliferation rates over multiple passages whereas the old donor cells grew progressively slower from week 5. Other studies have shown that MSC from old donors exhibit a decreased maximal life span and accelerated signs of senescence when compared with young donor cells [34]. In contrast, in another study there was no clear association between the maximal number of population doublings and donor age [35]. We observed that the old donor MSC became more granular and adopted a fried egg like and flatter larger morphology after time in culture, starting at passage 5–6; these morphological changes are also associated with replicative senescence [36]. In order to obtain a suitable number of cells for clinical transplantation it is likely that MSC would have to be expanded for multiple passages and then differentiated to the required phenotype. Previous studies have shown that adipocyte and osteogenic differentiation potential is decreased with passage number [37], [38] whereas neuronal differentiation does not appear to be significantly affected [39]. Multiple passaging of cells also has been suggested to mimic the aging process in vivo. For instance, proliferation rates are reduced and cells undergo senescence as a result of changes in expression levels of important cell cycle genes [36] and differentiation potential is reduced [37], [38] in cells grown under prolonged culture times; all these phenomena have been reported in cells isolated from old donors [24], [34], [40]. Importantly in our experiments we found that glial growth factor differentiation of MSC at late passage produced a similar response to cells treated at T3, i.e. further enhanced neurite outgrowth with young but not old donor MSC.

In summary our results indicate that MSC from young and old donors express significant levels of neurotrophic factors. Treatment of MSC with glial growth factors can boost the levels of BDNF and VEGF but enhanced DRG neurite outgrowth is only achieved with young donor MSC suggesting that other neurotrophic factors play a role. Time in culture does not appear to influence this process indicating that MSC from young donors could be successfully expanded and differentiated in suitable numbers for transplantation and treatment of nerve injuries.

## Supporting Information

Table S1 Primer sequences for RT-PCR with annealing temperatures (°C) and cycle number used.
(DOC)
