# Supplementary material for: Aging Effect on Neurotrophic Activity of Human Mesenchymal Stem Cells
Source: PLoS One. 2012 Sep 17;7(9):e45052. doi: 10.1371/journal.pone.0045052 (PMC3444498; doi:10.1371/journal.pone.0045052)
Supplement: Table S1 — Primer sequences for RT-PCR with annealing temperatures (°C) and cycle number used. (DOC) [file pone.0045052.s001.doc]

**Table 1.** Primer sequences for RT-PCR with annealing temperatures (˚C) and cycle number used.

| **Factor** | **Forward Primer (5’→3’)** | **Reverse Primer (5’→3’)** | **˚C** | **Cycles** |
| --- | --- | --- | --- | --- |
| S100 | GGAAATCAAAGAGCAGGAGGT | ATTAGCTACAACACGGCTGGA | 55.2 | 35 |
| GAPDH | GAAGGTGAAGGTCGGAGT | CAAGCTTCCCGTTCTCAGC | 62.0 | 35 |
| NGF | ATACAGGCGGAACCACACTCAG | GTCCACAGTAATGTTGCGGGTC | 63.7 | 32 |
| BDNF | AGAGGCTTGACATCATTGGCTG | CAAAGGCACTTGACTACTGAGCATC | 63.7 | 29 |
| GDNF | CACCAGATAAACAAATGGCAGTGC | CGACAGGTCATCATCAAAGGCG | 55.6 | 35 |
| NT3 | GGGAGATCAAAACGGGCAAC | ACAAGGCACACACACAGGAC | 61.6 | 32 |
| VEGF | TACCTCCACCATGCCAAGT | TGCATTCACATTTGTTGTGC | 53.5 | 29 |
